# Supplementary material for: FoxG1 regulates the formation of cortical GABAergic circuit during an early postnatal critical period resulting in autism spectrum disorder-like phenotypes
Source: Nat Commun. 2021 Jun 18;12:3773. doi: 10.1038/s41467-021-23987-z (PMC8213811; doi:10.1038/s41467-021-23987-z)
Supplement: Supplementary file 1 — Supplementary Information [file 41467_2021_23987_MOESM1_ESM.pdf]

Supplementary Materials

***FoxG1* regulates the formation of cortical GABAergic circuit during an early postnatal critical period resulting in autism spectrum disorder-like phenotypes**

Supplementary Figure 1 (related to Figure 2)
Supplementary Figure 2 (related to Figure 3)
Supplementary Figure 3 (related to Figures 1-3)
Supplementary Figure 4 (related to Figures 3)
Supplementary Figure 5 (related to Figures 3 and 4)
Supplementary Figure 6 (related to Figure 4)
Supplementary Figure 7 (related to Figure 6)
Supplementary Figure 8

Supplementary Figure 1

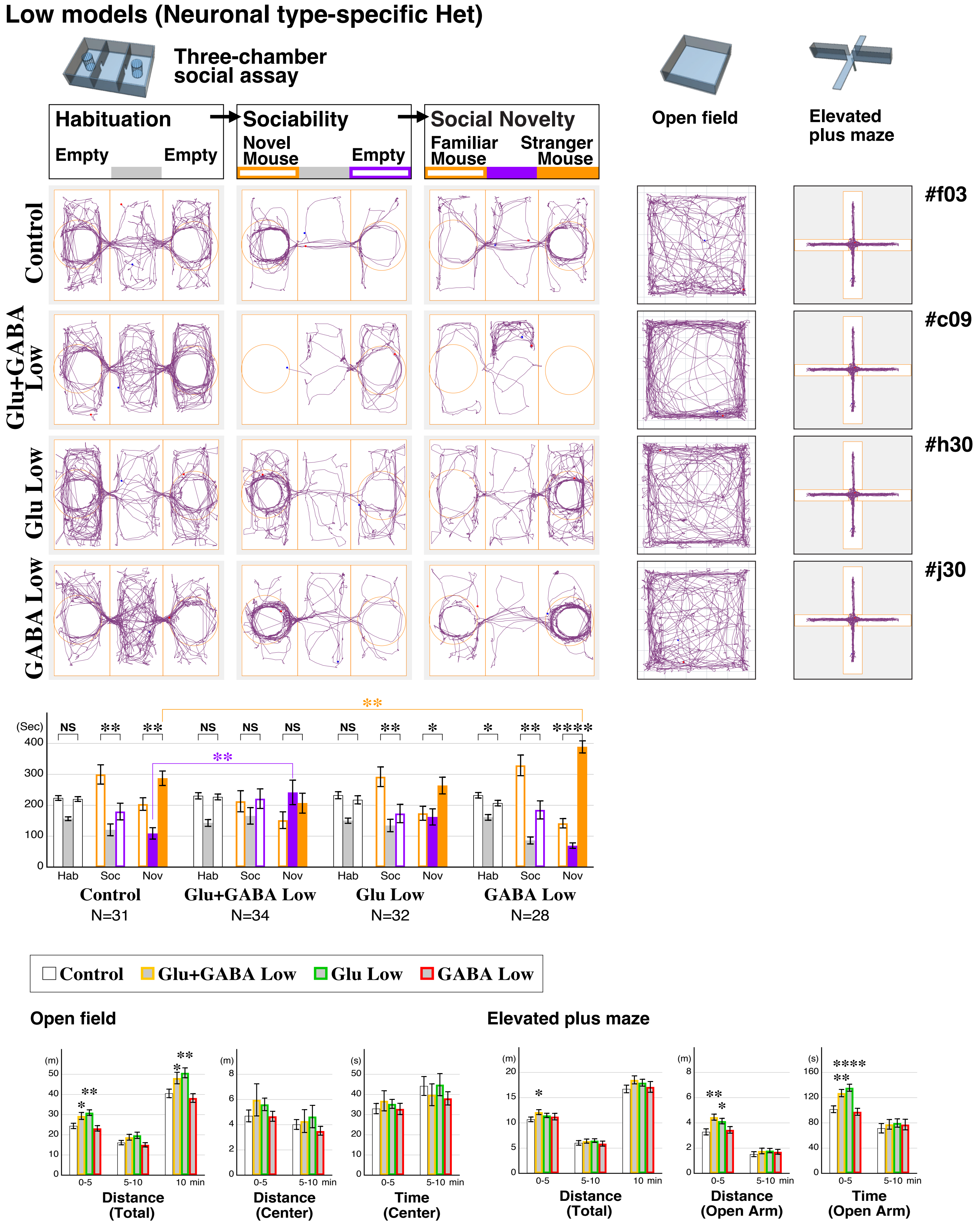

Supplementary Figure 1 (Related to Figure 2)

Behavior assays of the neuronal postmitotic *FoxG1* Low models (*floxed-FoxG1*, *Nex-Cre*, *Dlx-Cre*)
Example traces of the model animals for the Control (#f03), Glu+GABA Low (#c09), Glu Low (#h30) and GABA Low (#j30) are shown for the three different assays.
Three-chamber assay: Representative example traces (top) and bar graphs (middle row). Data are mean ± SEM and p values are from two-tailed *t*-test. Left vs Right chambers: Control(N=31): p=0.756(Hab), =0.00504**(Soc), =0.00937**(Nov), Glu+GABA Low(N=34) p=0.779(Hab), =0.858(Soc), =0.195(Nov), Glu Low(N=32): p=0.395(Hab), =0.00820**(Soc), =0.0135*(Nov), GABA Low(N=28): p=0.0490*(Hab), =0.00203**(Soc), =1.22x10(-13)****(Nov), Center chamber of Nov: Control vs Glu+GABA Low p=0.00401**, Social-side (Right, orange) chamber of Nov: Control vs GABA Low p=0.00154**
Open field: Example traces (top) and bar graphs (bottom left). Note that although there is no obvious change in the distance and time spent in the center for the Glu+GABA Low model, this particular animal #c09 preferred not to stay in the middle (20.2 sec in first 5min, 4.5 sec in last 5min). Glu Low and Glu+GABA Low models showed increased locomotion in this assay. Data are mean ± SEM and p values are from two-tailed *t*-test. p=0.0264*, =0.00108**, =0.529(0-5min, Distance total, left to right), =0.148, =0.0641, =0.482(5-10min), =0.0355*, =0.00249**, =0.452(10min), =0.354, =0.183, =0.959(0-5min, Distance center), =0.787, =0.520, =0.324(5-10min), =0.491, =0.491, =0.972(0-5min, Time), =0.543, =0.927, =0.287(5-10min)
Elevated plus maze: Example traces (top) and bar graphs (bottom right). Consistent with the open field assay, Glu+GABA Low model showed an increase in locomotion. Both Glu+GABA and Glu Low models traveled longer and spent more time on the open arm during the initial 5min, suggesting that these two models are initially less anxious, in a similar manner as the heterozygous model (Figure 1). Data are mean ± SEM and p values are from two-tailed *t*-test. p=0.0340*, =0.222, =0.493 (0-5min, Distance total, left to right), =0.565, =0.439, =0.833(5-10min), =0.108, =0.227, =0.760(10min), =0.00106**, =0.0107*, =0.659(0-5min, Distance Open arm), =0.401, =0.289, =0.499(5-10min), =0.00181**, =4.16x10(-5)****, =0.604(0-5min, Time), =0.560, =0.404, =0.603(5-10min)

Supplementary Figure 2

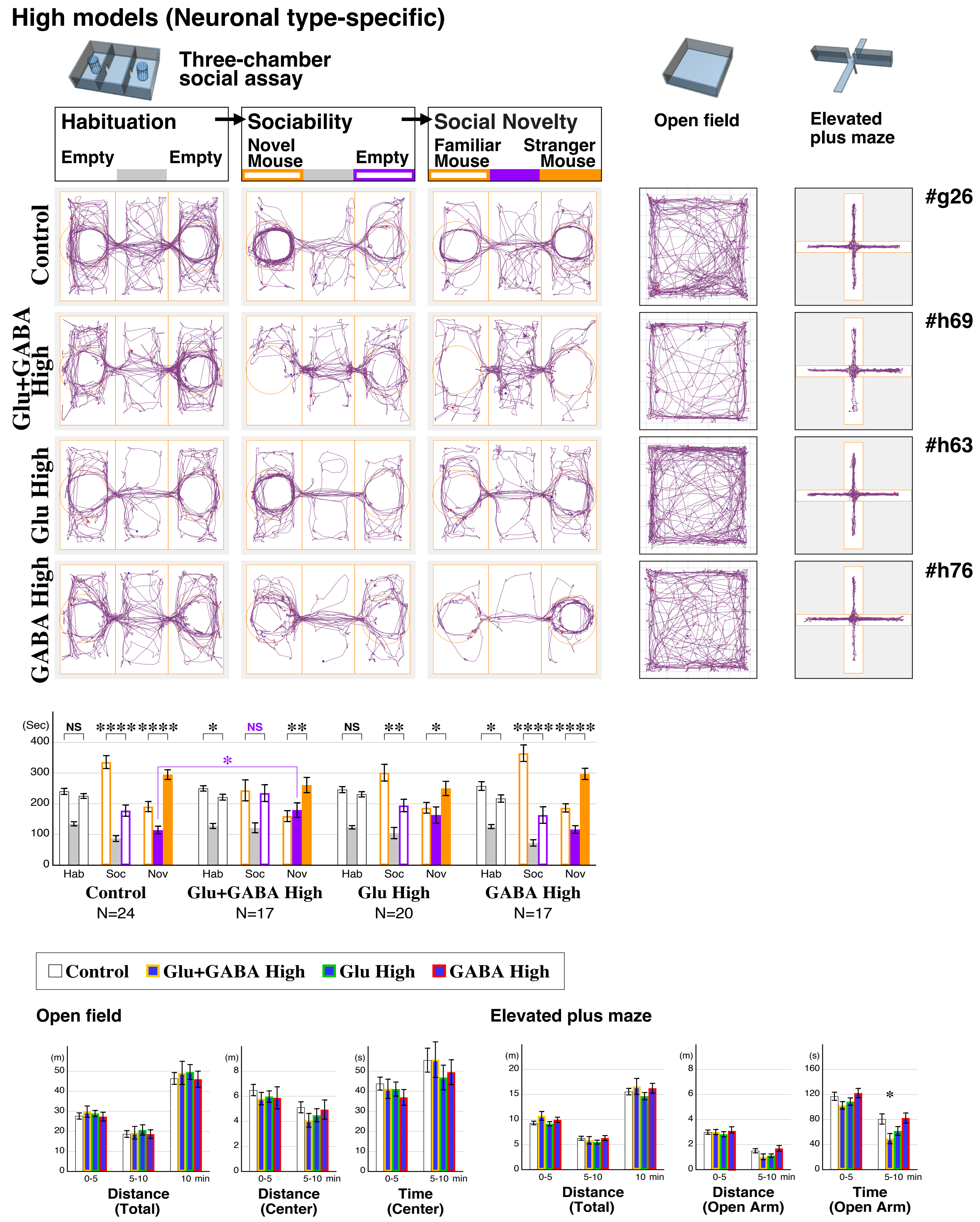

Supplementary Figure 2 (Related to Figure 3)

Behavior assays of the postnatal *FoxG1* High models (*TRE-FoxG1*, *R26-stop-tTA*, *Nex-Cre*, *Dlx-Cre*) Example traces of the model animals for the Control (#g26), Glu+GABA High (#h69), Glu High (#h63) and GABA High (#h76) are shown for the three different assays.

Three-chamber assay: Representative example traces (top) and bar graphs (middle row). Data are mean ± SEM and p values are from two-tailed *t*-test. Left vs Right chambers: Control(N=24) p=0.236(Hab), =8.53x10(-7)****(Soc), =3.58x10(-5)****(Nov), Glu+GABA High(N=17) p=0.0353*(Hab), =0.839(Soc), p=0.00254**(Nov), Glu High(N=20) p=0.249(Hab), =0.00330**(Soc), p=0.0358*(Nov), GABA High(N=17) p=0.0368*(Hab), =8.55x10(-6)****(Soc), p=3.47x10(-5)****(Nov), Center chamber of Nov: Control vs Glu+GABA High p=0.0231*

Open field: Example traces (top) and bar graphs (bottom left). We found no differences between the High models. Data are mean ± SEM and p values are from two-tailed *t*-test. p=0.508, =0.576, =0.907(0-5min, Distance total, left to right), =0.862, =0.515, =0.978(5-10min), =0.670, =0.497, =0.938(10min), =0.316, =0.419, =0.536(0-5min, Distance center), =0.149, =0.356, =0.838(5-10min), =0.657, =0.572, =0.187(0-5min, Time), =0.979, =0.320, =0.490(5-10min)

Elevated plus maze: Example traces (top) and bar graphs (bottom right). Glu+GABA High models spent less time on the open arm in the second 5min, suggesting that they become more anxious over time. Data are mean ± SEM and p values are from two-tailed *t*-test. p=0.136, =0.808, =0.317(0-5min, Distance total, left to right), =0.636, =0.176, =0.957(5-10min), =0.551, =0.361, =0.564(10min), =0.974, =0.515, =0.561 (0-5min, Distance Open arm), =0.107, =0.0911, =0.518(5-10min), =0.110, =0.336, =0.608(0-5min, Time), =0.0103*, =0.0839, =0.892(5-10min)

Supplementary Figure 3

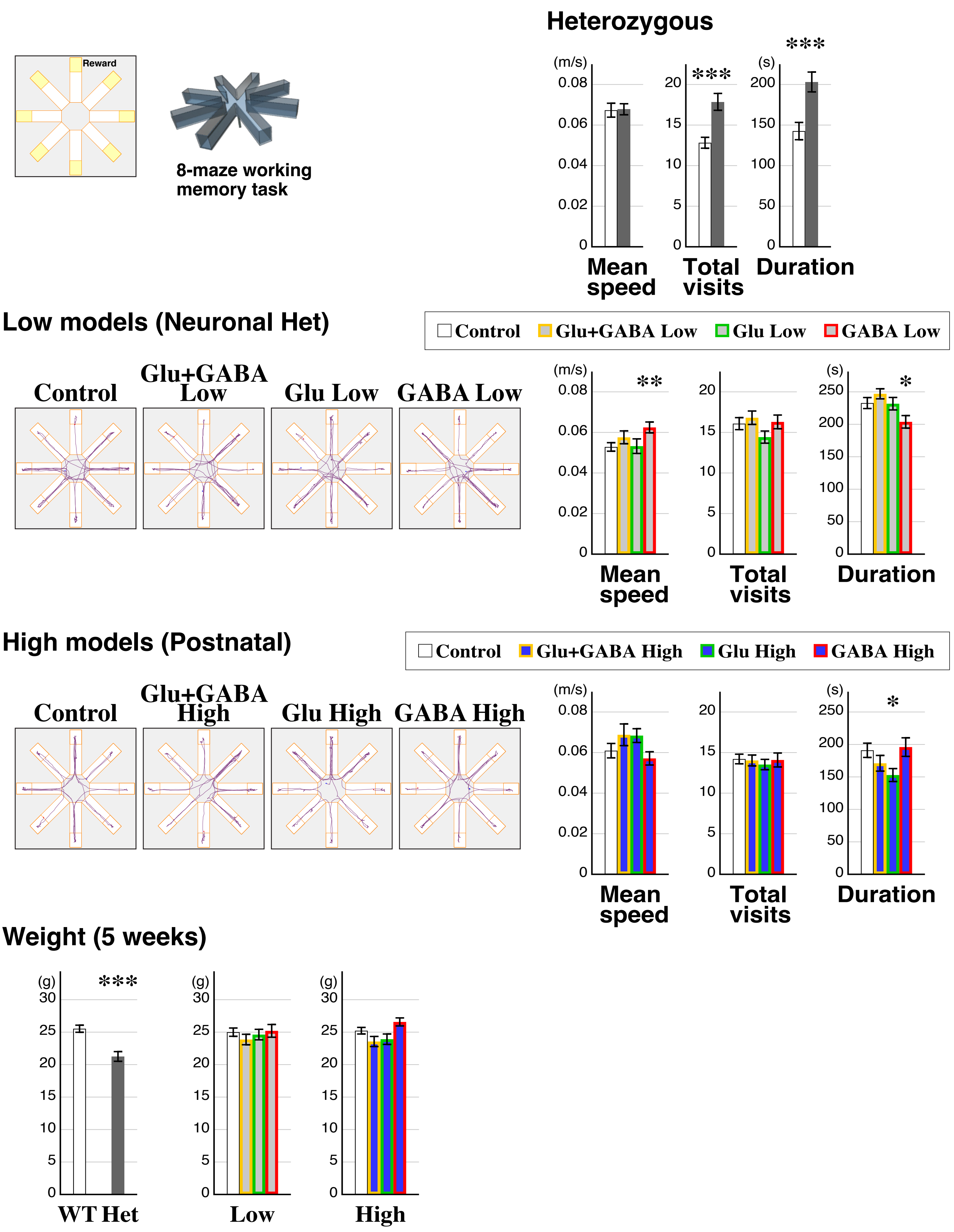

Supplementary Figure 3 (Related to Figures 1 to 3)

Working memory and weight of *FoxG1* Heterozygous, Low and High models

8-arm radial maze working memory task: Representative examples of traces (left) and bar graphs (right). The overall speed of GABA Low model animals was faster compared to Control, and animals took less time to finish the task, which is to collect the water droplet reward in all of the arms of the 8-arm radial maze. Total arm visits of this model were comparable to Control animals, indicating that working memory was unaffected. In a similar manner, Glu High animals completed the task faster than Controls, although the number of error visits made were comparable (Total visits). Thus, none of our postmitotic *FoxG1* expression manipulations exhibited altered working memory in the 8-arm radial maze. Data are mean ± SEM and p values are from two-tailed *t*-test. Heterozygous model (n=28, 28): p=0.916 (Mean speed), =0.000180***(Total visits), =0.000468***(Duration), Low models(n=62, 68, 64, 54, from left to right): p=0.215, =0.924, =0.00554**(Speed), =0.513, =0.118, =0.848(Visits), =0.218, =0.938, =0.0264*(Duration), High models(n=48, =34, =40, =34): p=0.228, =0.134, =0.432(Speed), =0.842, =0.435, =0.909(Visits), =0.225, =0.0119*, =0.784(Duration)

Animal weight: The weight of each animal at 5 weeks was recorded immediately after the open field assay. Heterozygous animals showed reduction in animal weight (n=14 each). No obvious difference was detected in the Low (n=20, 24, 21, 18) and High (n=24, 17, 20, 17) model animals. Data are mean ± SEM and p values are from two-tailed *t*-test. p=0.000110***(Het), p=0.276, =0.717, =0.865 (Low, from left to right), =0.0835, =0.177, =0.102(High)

Supplementary Figure 4

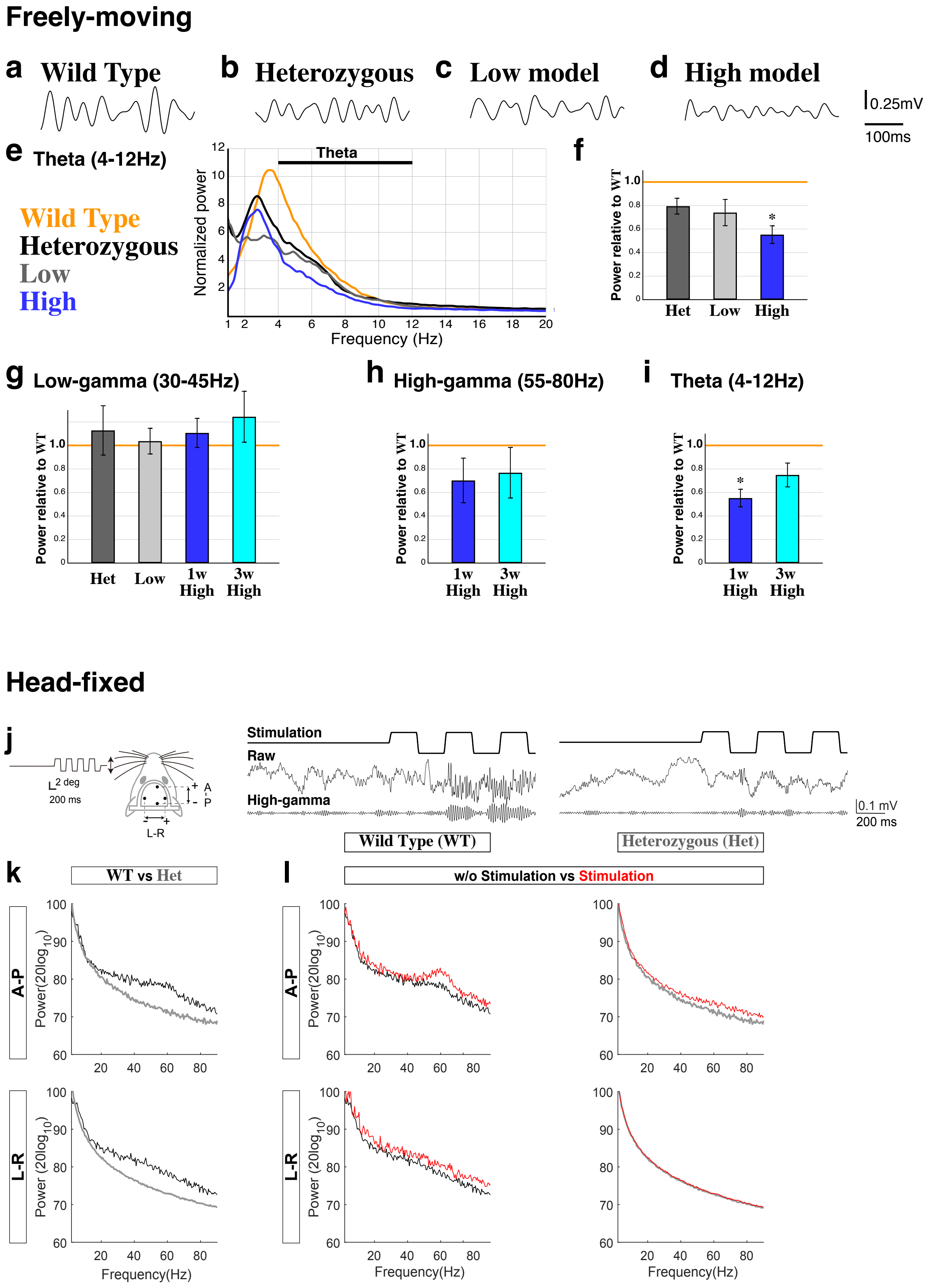

Supplementary Figure 4 (Related to Figure 3)

EEG analysis in freely-moving animals, or upon whisker stimulation in head fixed animals

(a-f) Analysis of theta band (4-12 Hz) EEG frequency of the Wild Type (a), Heterozygous (b), Glu+GABA Low (c) and Glu+GABA High (d) mouse models. High model showed reduction in the power of theta frequency (e, f, p=0.0485*) (g-i) Similar EEG analysis for the 3 week High model (n=3). 3 week High model with no obvious social impairments (Figure 4a) basically showed no changes in the EEG frequency powers compared to the Wild Type. All of the *FoxG1* models examined showed no changes in the power of low-gamma frequency range between 30-45 Hz (g). Comparisons in high-gamma and theta frequency ranges (h, i).

(j-l) EEG recordings during whisker stimulation was carried out by placing a piezoelectric device near the whiskers of the head-fixed animals. Raw and high-gamma (55-80Hz) filtered traces are shown at before and after the whisker deflection stimulation (j). EEG comparisons of Wild Type (n=3) and Heterozygous (n=3) animals at a rest state recorded from the electrodes placed between the anterior-posterior (top) or left-right positions (bottom, l). EEG comparisons of stimulation vs rest (without stimulation) state in Wild Type and Heterozygous animals (l). Heterozygous animals show very little response upon stimulation compared to the control animals.

Supplementary Figure 5

Western Blotting

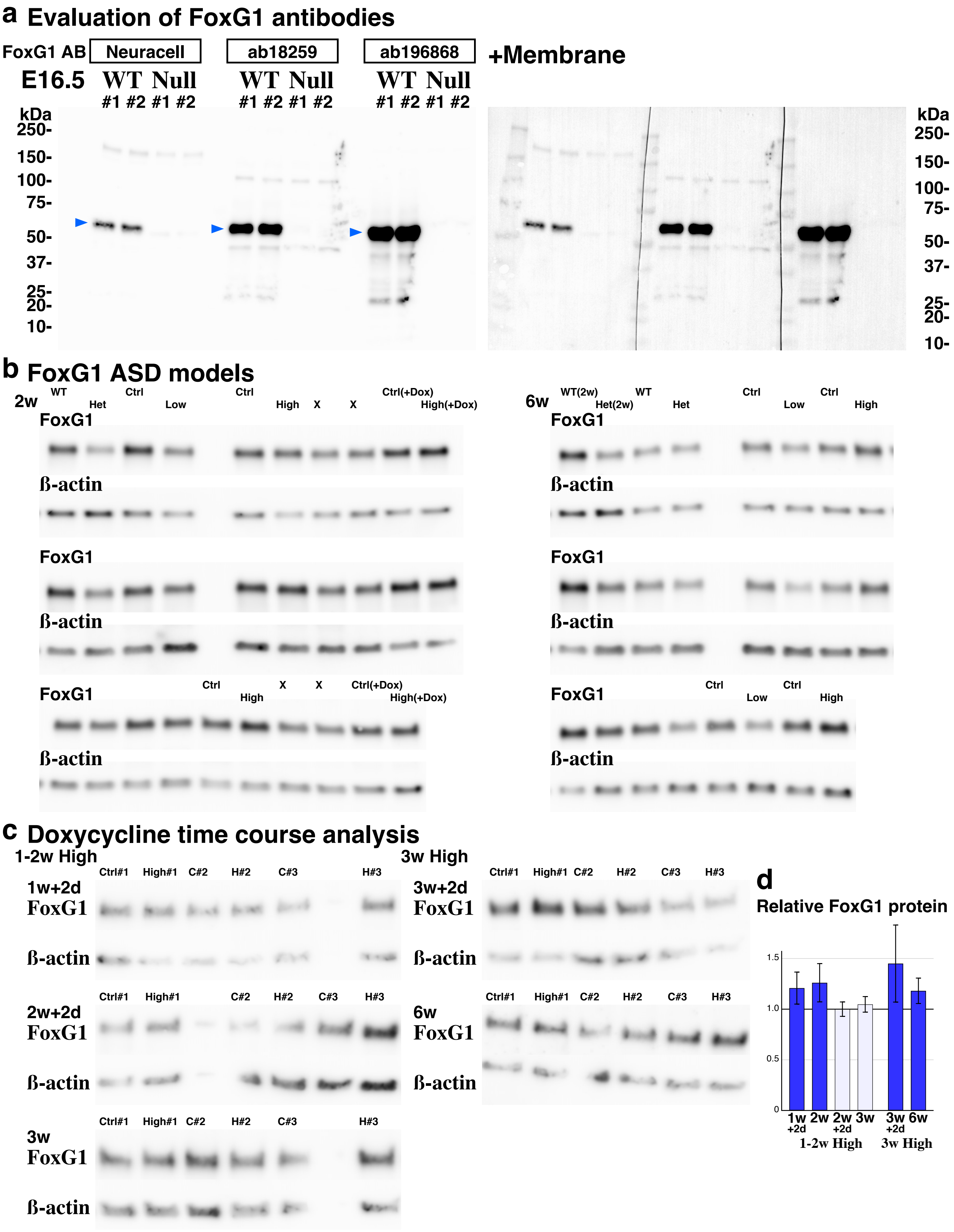

Supplementary Figure 5 (Related to Figures 3 and 4)

Western blotting analysis of the *FoxG1* ASD models

(a) Three commercially available FoxG1 antibodies were compared by using wild type (WT) and *FoxG1* Null embryonic 16.5 cortical tissues. Because of the non-specific bands found in the null tissues for the Neuracell and ab18259, we decided to use the ab196868 rabbit monoclonal antibodies for our study. (b) Western blotting raw data for Figures 3 m to 3o. (c, d) Western blotting analysis to identify the time course of doxycycline regulated *FoxG1* augmentation strategies. Raw data (c, n=3 each) and bar graphs (d) are shown. We conclude that *FoxG1* augmentation is induced or suppressed within two days after doxycycline withdrawal or administration to the animals.

Supplementary Figure 6

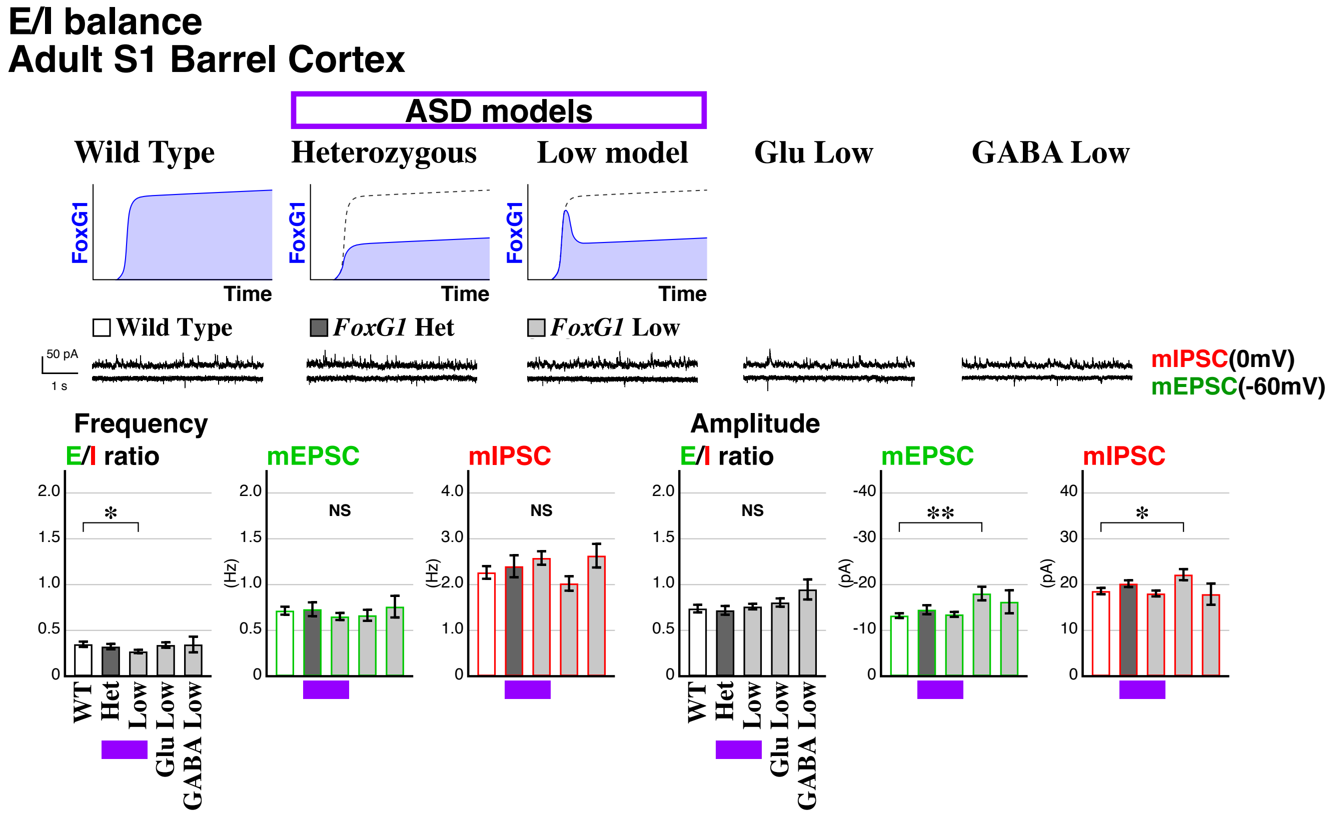

Supplementary Figure 6 (Related to Figure 4)

Excitatory-inhibitory ratio analysis in the adult somatosensory barrel cortex

Top: Representative traces of the miniature-excitatory and inhibitory postsynaptic currents (mEPSC and mIPSC) from a single layer 2/3 pyramidal cell in the presence of 0.5µM tetrodotoxin are shown for each *FoxG1* genetic model. No changes in the E/I ratio were observed in any of the models examined. Miniature events were obtained from Wild Type (n=28), *FoxG1* Heterozygous (n=11), Glu+GABA Low (n=29), Glu Low (n=17), and GABA Low (n=10) models. Excitation/inhibition (E/I) ratios of frequency, peak amplitude, and charge of miniature events. p=0.542, 0.0279*, 0.841, 0.978, =0.862, 0.280, 0.500, 0.735, =0.633, 0.124, 0.259, 0.231, =0.765, 0.675, 0.284, 0.100, =0.265, 0.724, 0.00642**, 0.268, =0.119, 0.582, 0.0158*, 0.787(from left to right). Data are mean ± SEM and p values are from two-tailed *t*-test.

Supplementary Figure 7

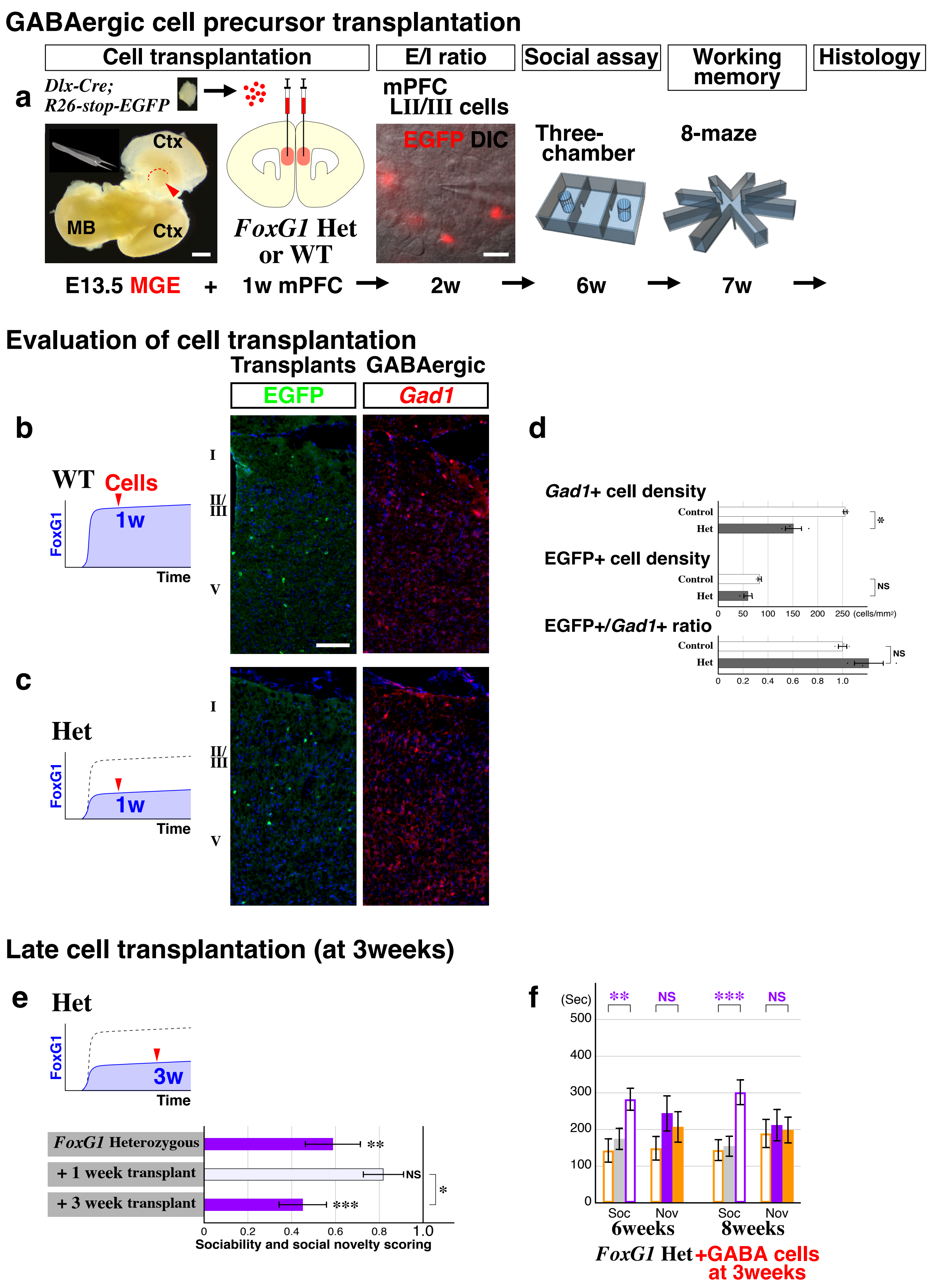

Supplementary Figure 7 (Related to Figure 6)

GABAergic neuronal precursor transplantation during the critical period ameliorates social phenotypes in the *FoxG1* ASD model

(a) In addition to the experimental scheme described in Figure 6a, we carried out 8-maze working memory task and histological analysis. (b-d) In order to evaluate the cell transplantation experiments, upon completing the working memory task, brains were fixed and EGFP-labeled MGE-derived GABAergic donor cell numbers (*Dlx-Cre*; *RCE:loxP*) and overall GABAergic cell numbers (*Gad1 in situ* hybridization) were both analyzed in adjacent sections (b, c). DAPI stain is shown in blue. Consistent with what we have found at three weeks of age (Figures 2a to 2k), overall *Gad1*+ cell density was reduced in the Heterozygotes compared to the controls (d). Donor-cell density per cortical area as well as per GABAergic cell numbers were comparable between the control and Heterozygous animals (n=3 each). Error bars are ± SEM, two-tailed *t*-test for Control vs. Het: p=0.0190*, =0.0870, =0.204 (d, top to bottom) (e, f) Late-transplanted (3 weeks) Heterozygous animals at 6 weeks showed ASD-like social behavioral alterations (Figure 6j). Sociability scores are compared between *FoxG1* Heterozygous (Figure 1) and 1- and 3-week cell transplanted Heterozygous models (e). 3-week transplanted animals (WT: n=32, Het: n=21) showed significantly decreased sociability (p=0.000116***) and were distinct from 1-week transplantation experiments (p=0.0129*). In order to match the differentiation period of the donor cells to the postnatal 1-week transplantation experiments, we analyzed late-transplanted animals at 8 weeks, instead of 6 weeks (f). Still, they were not like the 1-week transplanted Heterozygotes, suggesting that cell transplantation after the critical period does not ameliorate the social behavior of the animals. Error bars are ± SEM, two-tailed *t*-test for 8 weeks: p=0.000943***(Soc), =0.858(Nov)

Supplementary Figure 8

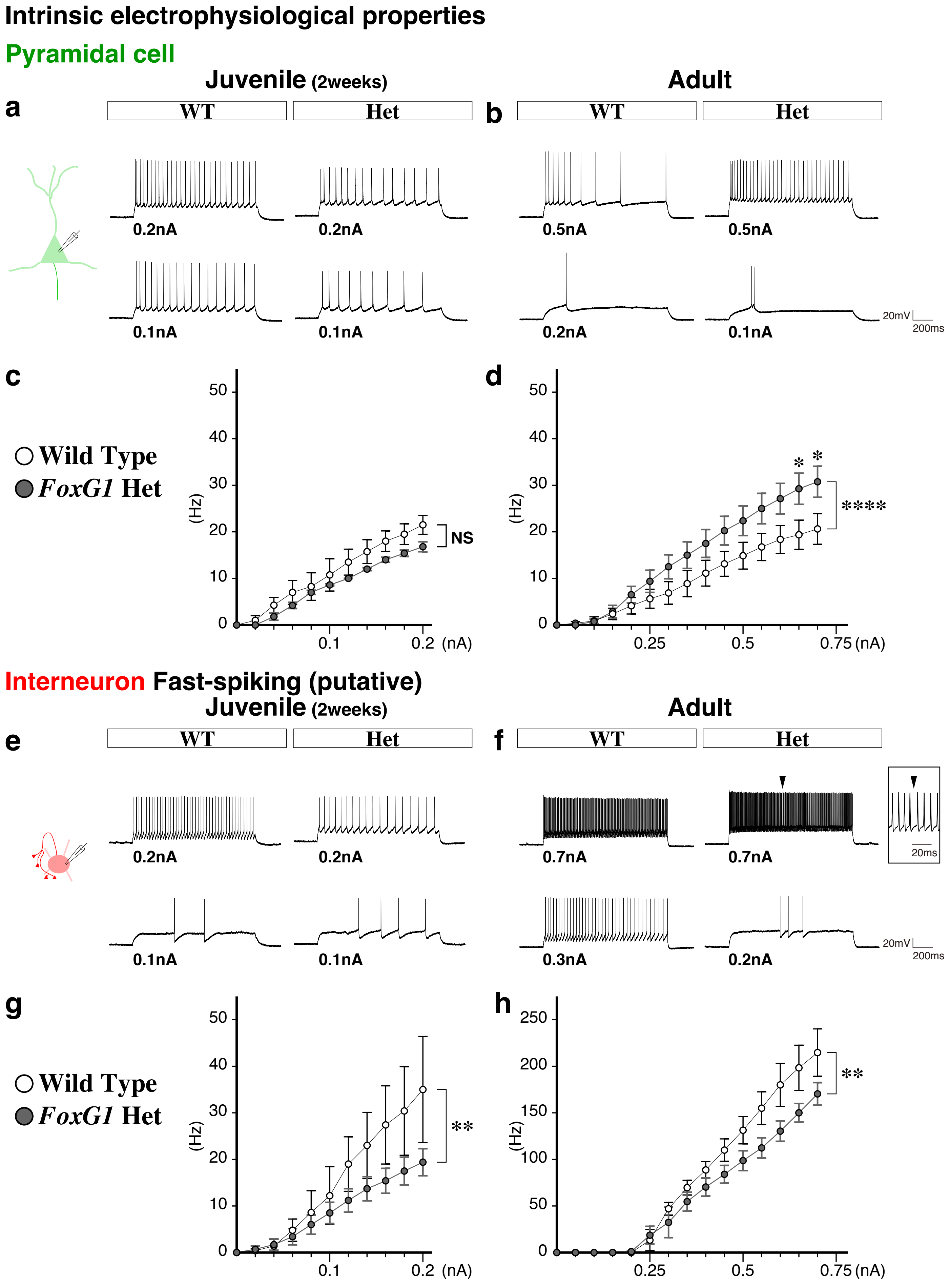

Supplementary Figure 8

Intrinsic electrophysiological properties of the mPFC neurons in *FoxG1* Heterozygous model

(a-d) Representative traces of the intrinsic firing properties of pyramidal cells in the layer 2 of the medial prefrontal cortex (prelimbic). Cells were recorded from juvenile (a, P13 or P14) and adult animals (b, 6 to 7 weeks). Het pyramidal cells often showed doublet action potentials as an initial response to the current injection in adults (b). These pyramidal cells with doublets are normally found in the layer 5/6 but not in the layer 2/3. In adults, Het pyramidal cells show reduced firing potentials based on the frequency-current analysis (c, d). n=4(c, WT), =5(Het) and =8(d, WT), =8(Het) p=0, 0.292, 0.195, 0.274, 0.650, 0.505, 0.195, 0.143, 0.0892, 0.0910, 0.0649 (c, left to right), = 0, 0.334, 0.904, 0.787, 0.360, 0.250, 0.133, 0.147, 0.140, 0.102, 0.102, 0.0807, 0.0691, 0.0491*, 0.0486* (d) Error bars are mean ± SEM, p values are from two-tailed *t*-test. Through two-way ANOVA and post-hoc Tukey's analysis of the frequency-current plot, wild type and Het pyramidal cells are found different in adults (p<0.0001****, d) but not in juvenile animals (p=0.117, c).

(e-h) Recordings from the layer 2 GABAergic cells, which are labeled by EGFP (*Dlx-Cre*; *RCE:loxP*). In order to collect data from putative fast-spiking cells at juvenile stages, neurons with a rebound-spike (presumably, low-threshold spiking subtype) and the ones with a pronounced voltage sag were excluded from the analysis (e). Adult Het fast-spiking cells often showed an irregular attenuation during the current injection (arrowheads, squared panel is a high-magnification, f). Frequency-current analysis results (g, h). n=5(g, WT), =10(Het) and =3(h, WT), =3(Het) p=0, 0.778, 0.882, 0.647, 0.561, 0.501, 0.170, 0.151, 0.104, 0.120, 0.0999 (g), =0, 0, 0, 0, 0.373, 0.737, 0.453, 0.307, 0.237, 0.165, 0.146, 0.107, 0.124, 0.139, 0.190 (h) Error bars are mean ± SEM, p values are from two-tailed *t*-test. Through two-way ANOVA and post-hoc Tukey's analysis of the frequency-current plot, Het putative fast-spiking interneurons are found different from the wild types at both juvenile (p=0.0039**, g) and adult stages (p=0.0014**, h).
